# Neural ensembles: role of intrinsic excitability and its plasticity

**DOI:** 10.3389/fncel.2024.1440588

**Published:** 2024-07-31

**Authors:** Christian Hansel, Rafael Yuste

**Affiliations:** ^1^Department of Neurobiology and Neuroscience Institute, University of Chicago, Chicago, IL, United States; ^2^NeuroTechnology Center, Department of Biological Sciences, Columbia University, New York, NY, United States

**Keywords:** engram, ensemble, excitability, intrinsic plasticity, learning, memory, memory allocation, synaptic plasticity

## Abstract

Synaptic connectivity defines groups of neurons that engage in correlated activity during specific functional tasks. These co-active groups of neurons form ensembles, the operational units involved in, for example, sensory perception, motor coordination and memory (then called an *engram*). Traditionally, ensemble formation has been thought to occur via strengthening of synaptic connections via long-term potentiation (LTP) as a plasticity mechanism. This synaptic theory of memory arises from the learning rules formulated by Hebb and is consistent with many experimental observations. Here, we propose, as an alternative, that the intrinsic excitability of neurons and its plasticity constitute a second, *non-synaptic* mechanism that could be important for the initial formation of ensembles. Indeed, enhanced neural excitability is widely observed in multiple brain areas subsequent to behavioral learning. In cortical structures and the amygdala, excitability changes are often reported as transient, even though they can last tens of minutes to a few days. Perhaps it is for this reason that they have been traditionally considered as modulatory, merely supporting ensemble formation by facilitating LTP induction, without further involvement in memory function (memory allocation hypothesis). We here suggest−based on two lines of evidence—that beyond modulating LTP allocation, enhanced excitability plays a more fundamental role in learning. First, enhanced excitability constitutes a signature of active ensembles and, due to it, subthreshold synaptic connections become suprathreshold in the absence of synaptic plasticity (*iceberg model*). Second, enhanced excitability promotes the propagation of dendritic potentials toward the soma and allows for enhanced coupling of EPSP amplitude (LTP) to the spike output (and thus ensemble participation). This *permissive gate model* describes a need for permanently increased excitability, which seems at odds with its traditional consideration as a short-lived mechanism. We propose that longer modifications in excitability are made possible by a low threshold for intrinsic plasticity induction, suggesting that excitability might be on/off-modulated at short intervals. Consistent with this, in cerebellar Purkinje cells, excitability lasts days to weeks, which shows that in some circuits the duration of the phenomenon is not a limiting factor in the first place. In our model, synaptic plasticity defines the information content received by neurons through the connectivity network that they are embedded in. However, the plasticity of cell-autonomous excitability could dynamically regulate the ensemble participation of individual neurons as well as the overall activity state of an ensemble.

## Introduction

Synaptic long-term potentiation (LTP; Bliss and Lømo, 1973) translates experiences via its activity-dependence into enhanced synaptic efficacy. LTP may also be accompanied by the emergence of new dendritic spines (Engert and Bonhoeffer, 1999; Yuste and Bonhoeffer, 2001) and spine growth (Holtmaat and Svoboda, 2009) making it the ideal plasticity mechanism to establish and update synaptic connectivity in an experience-dependent manner in neural ensembles. The ultimate proof for a causal relationship between synaptic plasticity and memory was provided in 2014, when Malinow et al. optogenetically induced long-term depression (LTD) and LTP to inactivate and reactivate, respectively, a fear memory that was previously established using a fear-conditioning paradigm in the amygdala (Nabavi et al., 2014). In fear-conditioning, a tone is often used as the neutral conditioned stimulus (CS), which is paired with an electric footshock as the unconditioned stimulus (US). Nabavi et al. replaced the tone presentation with optical stimulation (channelrhodopsin 2; ChR2) of axons in an auditory relay nucleus that projects to the amygdala. Application of LTD- and LTP- inducing stimuli (tested separately *in vivo*), respectively, was sufficient to disconnect/connect the CS pathway to the fear memory engram in the amygdala. This is a critical finding due to the immediate optical control of synaptic weight and its impact on behavioral learning.

However, conditioning experiments have revealed a second parameter that changes with learning and might be causally related to engram formation as well. *Ex vivo* recordings from CA1 hippocampal pyramidal neurons following eyeblink conditioning revealed that the intrinsic membrane excitability was enhanced in neurons from conditioned, but not pseudoconditioned or naïve rabbits (Disterhoft et al., 1986). Similar findings were made previously following associative learning in the mollusk *Hermissenda* (Alkon, 1984) and subsequently following eyeblink conditioning in cerebellar Purkinje cells (Schreurs et al., 1998). In line with these and other findings, it has been suggested that there could be a “Memory from the dynamics of intrinsic membrane currents” (Marder et al., 1996), an idea that was followed up by several investigators soon after (Hansel et al., 2001; Daoudal and Debanne, 2003; Frick et al., 2004). How have these ideas been implemented into modern theories of memory? Surprisingly, intrinsic plasticity plays a relatively minor role in current learning models. In the memory allocation hypothesis, the learning model with closest focus on cellular mechanisms of engram formation and recall, neurons are allocated to an engram that show high excitability at the time of learning, facilitating subsequent integration via LTP (Rogerson et al., 2014; Yiu et al., 2014; Josselyn and Frankland, 2018; Josselyn and Tonegawa, 2020). Perhaps the disregard for a deeper involvement of intrinsic plasticity stems from the observation that excitability changes are often short-lived. A memory engram related to fear-conditioning in the dentate gyrus was characterized by a state of enhanced neuronal excitability, but this effect faded within two hours (Pignatelli et al., 2019). In prior *ex vivo* recordings from CA3 hippocampal pyramidal neurons following eyeblink conditioning in rabbits, enhanced excitability was observed for longer periods of time, but began to decline three days after learning (the conditioned behavior, in contrast, lasted for the full 180 days of recordings; Thompson et al., 1996). These findings seem to suggest that enhanced excitability cannot play a more permanent role in engram physiology, for example in memory recall. Here, we argue that despite of its transient nature enhanced excitability via intrinsic plasticity is necessary and, in some scenarios sufficient, for the formation and reactivation of ensembles in general, including ensembles that serve as memory engrams.

## Enhanced excitability in cortical ensembles: the iceberg model

As mentioned, neuronal ensembles (often referred to as assemblies) are coactive groups of neurons that have been shown to mediate perception and behavior (Buzsaki, 2010*;* Yuste et al., 2024). In mouse primary visual cortex, ensembles are formed by a small group (∼10%) of imaged neurons, which become coactive in a significant manner, within a small temporal window (Cossart et al., 2003: Miller et al., 2014; Carrillo-Reid et al., 2015). Neurons can participate in several ensembles, demonstrating that ensembles can form a multineuronal code (Miller et al., 2014). Indeed, ensembles are formed by both neurons that are specifically co-activated (onsembles) as well as neurons that are specifically silenced (offsemble), further indicating that they generate an emergent code (Pérez-Ortega et al., 2024). Offsemble neurons have shorter calcium decay kinetics than onsemble cells, suggesting that their activity is curtailed by GABAergic inhibition. Different classes of interneurons may dynamically control dendritic and somatic compartments of target pyramidal neurons. Interneuron activity itself may be controlled by synaptic and intrinsic plasticity mechanisms. Individual ensembles are activated by visual stimuli, both moving gratings and naturalistic videos. Activity in similar groups of neurons can be observed in the absence of external stimuli (ongoing activity), as if these circuits were internal building blocks of cortical activity that can be allocated to become engrams. This interpretation is supported by the observation that these ongoing ensembles have statistically the same neuronal components as the ones activated by visual stimuli (Miller et al., 2014). In addition, some cortical ensembles persist for up to 46 days, albeit with a substantial rotation of individual neuronal participants anchored by a group of more persistent core neurons (Perez-Ortega et al., 2021). The general feature of (relative) permanency is consistent with the possibility that ensembles serve as repositories of perceptual and memory states. In agreement with this, the optogenetic reactivation of ensembles can recall previous visual stimuli, and lead to a behavioral outcome (Carrillo-Reid et al., 2019; Marshel et al., 2019). Thus, ensembles are not an epiphenomenon of cortical activity but are causally linked to perceptual states (Carrillo-Reid et al., 2017).

Interestingly, cortical ensembles can also be created *de novo*: optogenetic co-activation of neurons leads to their joint ongoing co-activation, forming a new ensemble which can last for days (Carrillo-Reid et al., 2016). This result can explain the concordance between visually evoked and ongoing ensembles: one can posit that sensory stimulation can imprint ensembles into the cortex and these ensembles can then be recalled internally, manifesting themselves later in the ongoing activity. Consistent with this, stimulus-evoked ensembles are recalled during sleep (Lines and Yuste, 2023). This result can also explain the concordance between ensembles and engrams, whereby engrams could represent the recalling of previously imprinted ensembles. Importantly, the optogenetics imprinting of ensembles by coactive activation of neurons initially strongly suggested that ensembles were generated by Hebbian synaptic plasticity, whereby neurons that fire together strengthen their synaptic connectivity.

**FIGURE 1:**
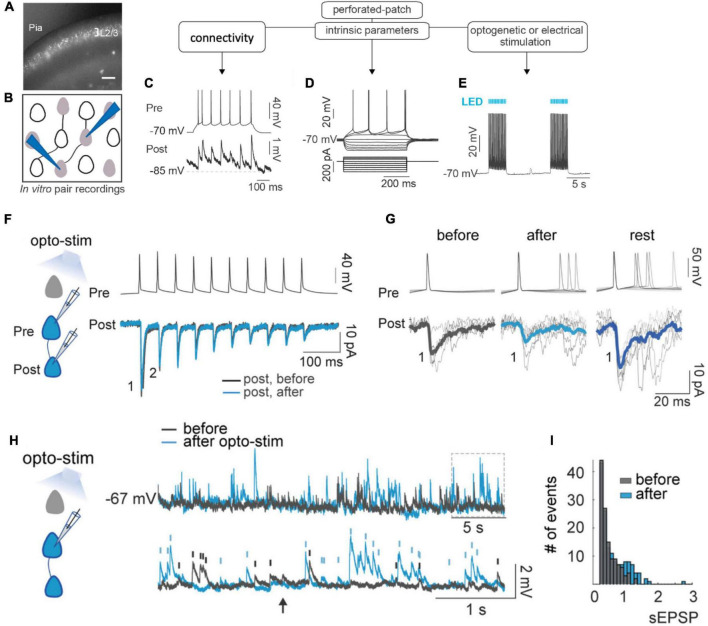
Ensemble formation leads to increased excitability. **(A)** Experimental design. Image of brain slice from mouse in primary visual cortex with ST-ChroMe opsin expression in pyramidal neurons. Scale bar: 200 μm. **(B)** Illustration of in-vitro paired recording for evaluating monosynaptic connectivity between neurons. **(C)** Perforated patch-clamp recording of presynaptic action potentials elicited by current injections (500 ms), followed by identification of a monosynaptic connection, generating postsynaptic potentials time-locked to presynaptic spikes. **(D)** Intrinsic parameters were tested, such as firing rate, input resistance, and firing threshold with the changes in membrane voltage in response to current steps. **(E)** Perforated current-clamp recording under optogenetic stimulation protocol: 1 to 30 min of 10 Hz train, 5 ms light pulses for 4 s followed by 10 s of rest. **(F)** Representative paired whole-cell recording of synaptically connected neurons. Top: current-clamp recording of presynaptic action potentials in response to 10 current injections (2 ms each; 20 Hz). Bottom: voltage-clamp recording of evoked EPSC before (black) and after (blue) 30 min of optogenetic stimulation. Each trace is average of 30 successive responses evoked by presynaptic current injection. **(G)** Representative paired recording of evoked EPSCs (perforated patch–clamp). Top: current-clamp recording of presynaptic action potentials induced by positive current steps of the I–V curve (20–120 pA). Bottom: voltage-clamp recording of evoked EPSCs before and after optogenetic stimulation and after 20 min of rest post-stimulation. Thick line is average of successive responses to the first presynaptic action potentials, for every current step of the I–V curve. **(H)** Increase in spontaneous activity after optogenetic stimulation. Representative perforated patch-clamp recording of a neuron in current-clamp before (black) and after (blue) optogenetic stimulation protocol. Bottom: Section in the top trace shows spontaneous EPSPs amplitude > 0.3 mV identified before (black) and after (blue) optogenetic stimulation protocol in a one-minute recording. Arrows show tentative EPSPs that were not detected by the threshold (0.3 mV). **(I)** Frequency histogram of EPSPs amplitudes shows that after optogenetic stimulation, the number and amplitude of spontaneous synaptic events increased. (Modified with permission from Alejandre-García et al., 2022).

To better understand the mechanisms that lead to the formation of ensembles, we co-activated optogenetically and electrically layer 2/3 pyramidal neurons in brain slices, replicating *in vitro* the optogenetic protocol to generate ensembles in vivo (Carrillo-Reid et al., 2016; Alejandre-García et al., 2022; Figures 1A–E). Using whole-cell and perforated patch-clamp pair recordings we found, to our surprise, that, after optogenetic or electrical stimulation, there were only minor changes in synaptic plasticity (Figures 1F–H). In fact, instead of synaptic potentiation, co-activated neurons actually showed an initial depression, followed by a small rebound potentiation after a recovery period. There was also no evidence that new connections formed after optogenetic stimulation in previously unconnected neurons (Alejandre-García et al., 2022). Thus, synaptic plasticity could not explain the emergence of neuronal ensembles in this protocol. But, unexpectedly, optogenetic and electrical stimulation induced major increases in amplitude and frequency of spontaneous EPSPs, even after single-cell stimulation (Figures 1I, J). Consistent with this, we observed strong and persistent increases in neuronal excitability after stimulation, along with increases in membrane resistance and reduction in spike threshold (Alejandre-García et al., 2022). Similar increases in ongoing activity had also been noticed after ensemble imprinting *in vivo* (Carrillo-Reid et al., 2016). We concluded that intrinsic excitability, rather than Hebbian plasticity, mediates the establishment of neuronal ensembles.

**FIGURE 2:**
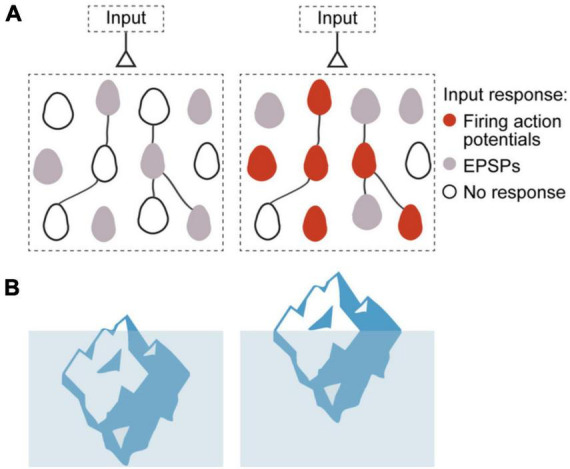
Iceberg model of ensemble formation. **(A)** Emergence of ensembles after increases in neuronal excitability, illustration circuit response before (left) and after (right) ensemble generation. Color corresponds to membrane potential response to a synaptic input: white is resting membrane potentials, gray are subthreshold responses, and red are suprathreshold ones, with firing of action potentials. Stimulated neurons become more excitable, so the same inputs induce some of them to fire (red cells), while other cells have increased subthreshold responses (gray cells). The model explains how an ensemble is formed but does not assume any changes in numbers or strength of local synapses. **(B)** Left: Iceberg emergence: An iceberg keeps underwater. Right: But if its density decreases, the iceberg emerges above water. Density is an intrinsic property of the iceberg and, by changing it, the iceberg changes its response to the same environment. Likewise, for a neuronal ensemble, membrane resistance and firing threshold are intrinsic neuronal properties that can be modified, and they enhance its response to the same excitatory input intensity, resulting in an increased depolarization and generation of action potentials (Modified with permission from Alejandre-García et al., 2022).

To explain how neuronal ensembles are generated, we propose an “iceberg” model, by which the increased neuronal excitability that results from repeated input stimulation makes subthreshold connections become suprathreshold, enhancing the postsynaptic effect of already existing synapses, and generating a neuronal ensemble (Figure 2; Alejandre-García et al., 2022). This increase in synaptic efficacy is in fact strictly consistent with the original Hebbian postulate, which relates to the effect that a synapse has on the postsynaptic cell (Hebb, 1949), but did not necessarily imply increase in synaptic transmission or strength.

## Permanent role for enhanced excitability despite transient nature: role for an on/off modulation

**FIGURE 3:**
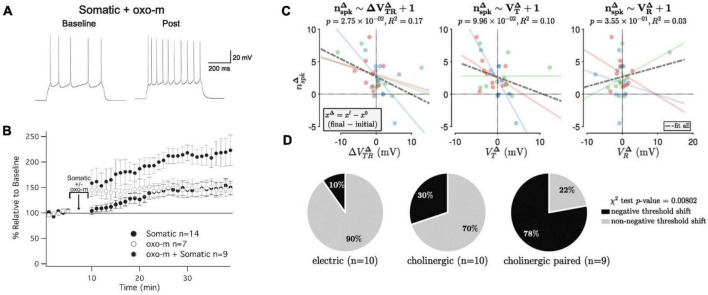
Cholinergic modulation promotes intrinsic plasticity in L2/3 pyramidal neurons of mouse primary somatosensory cortex (S1). **(A)** Example recording of a neuron that received somatic depolarization (10 Hz; 5 min) while the muscarinic agonist oxotremorine-1 (oxo-m; 7 μM) is applied to the bath during these *in vitro* recordings. **(B)** Time graph showing changes in spiking relative to baseline; the three conditions shown are oxo-m bath-application alone, somatic depolarization alone, and both stimuli combined. **(C)** Cholinergic modulation shifts the neuronal threshold potential. Relationship between the functional change (number of evoked spikes; n_spk_) and the membrane potential change (V_R_ = resting potential; V_T_ = threshold potential; V_TR_ = difference between the two potentials). Blue dots: electric stimulation alone; green dots: cholinergic modulation alone; red dots: paired stimulation. There is a significant negative association between the changes in spike number and threshold-to-rest distance changes, but not with changes in the threshold or resting potential changes. **(D)** Distribution of S1 recordings from the three experimental groups according to category membership, based on δ V_TR_, to either ‘negative threshold shift’ or ‘non-negative threshold shift’ categories. Only paired electric and cholinergic activation causes a significant negative threshold shift. **(A,B)** are taken from Gill and Hansel (2020), *eNeuro* 7. **(C,D)** are taken from Pham and Hansel (2023), *J. Physiol*. 601.15.

Let us assume for a moment that enhanced neuronal excitability is essential for ensemble function (in a later paragraph, we will present evidence to support this claim). Would not the transient nature of excitability potentiation make such essential contribution impossible? To begin with, it should be noted that intrinsic plasticity is not in all brain structures and neuron types short-lived. In cerebellar Purkinje cells, enhanced excitability was observed one month after eyeblink conditioning (Schreurs et al., 1998). This finding suggests that there could be more cell types, in which intrinsic plasticity is long-lasting. It further suggests the possibility that the underlying mechanism is the same or similar in different cell types, with the potential for a long effect duration, but that in some and not others specific activity patterns curtail duration. The second, perhaps more general, point is that enhanced excitability does not need to last permanently to play a permanent role in ensemble function as long as it can be readily recruited under the right conditions (on/off modulation). This criterion is fulfilled when a (transient) excitability increase results from attention-related signaling. Indeed, as illustrated in Figure 3, intrinsic plasticity is evoked in cortical pyramidal neurons upon activation of muscarinic acetylcholine receptors (mAChRs).

Intrinsic plasticity in L5 pyramidal neurons (Sourdet et al., 2003), L2/3 pyramidal neurons (Gill and Hansel, 2020) and cerebellar Purkinje cells (Belmeguenai et al., 2010) depends on the downregulation of small-conductance calcium-dependent SK-type K^+^ channels. In L5 pyramidal neurons, this downregulation was driven by activation of type 5 metabotropic glutamate receptors (mGluR5; Sourdet et al., 2003), while in L2/3 pyramidal neurons, it was achieved by activation of mAChRs (Gill and Hansel, 2020; Figure 3). Both are Gαq-coupled metabotropic receptors. For M1 mAChRs, it has indeed been demonstrated that their activation inhibits SK-type K^+^ channels (Buchanan et al., 2010; Giessel and Sabatini, 2010). These findings show that cholinergic signaling may promote enhanced excitability via the inhibition of SK channels.

**FIGURE 4:**
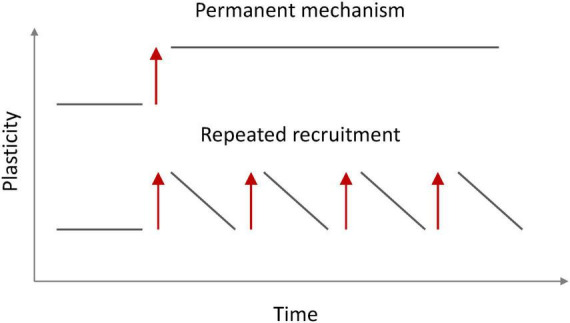
Ensemble availability for recall via permanent (top) and non-permanent (bottom) on/off modulation. A synaptically connected ensemble does not require a permanent plasticity mechanism to enhance the probability for recall as long as the ensemble can be readily reactivated (red arrows). This activation involves synaptic drive, but not synaptic plasticity.

The observation that intrinsic plasticity may be triggered by cholinergic signaling is important for our claim that there is no strict need for permanency in ensemble plasticity (as long as the participating neurons are synaptically connected). We postulate two requirements for a non-permanent mechanism that instead rests on the availability to readily re-activate the ensemble and thus be on/off-modulated (Figure 4). First, ensemble re-activation needs to be triggered by a ‘meaningful’ signal; that is to say that a cellular mechanism should link ensemble re-activation to a context that can be expected to recruit and engage ensembles. Cholinergic signaling fulfills this requirement as it occurs in the context of attention (Everitt and Robbins, 1997). Second, a mechanism for ensemble re-activation would need to be readily available and fast without affecting synaptic connectivity. This is the case for intrinsic plasticity, which−at least in the hippocampus−has been shown to have a lower induction threshold than synaptic plasticity (Lopez-Rojas et al., 2016). These considerations show that−once a second plasticity mechanism comes into play−there is no need for one mechanism whose long-duration (permanency) would guarantee longevity of memory itself. Instead, one plasticity mechanism (synaptic) would establish connectivity and ensemble identity but does not need to be further modulated upon re-activation, while the other (intrinsic) enhances excitability and ensures ensemble function as long as required. On/off modulation as described in Figure 4 furthermore guarantees that flexibility in the activation of various−potentially competing−ensembles is not jeopardized. In addition, intrinsic plasticity can be depressed in an activity-dependent manner (e.g., Paz et al., 2009) and this bidirectionality prevents saturation.

## Role of enhanced excitability in ensemble formation and function

The memory allocation hypothesis describes a transient role for intrinsic plasticity in the formation of memory engrams. In this conceptual framework, enhanced excitability promotes LTP induction and thus contributes to engram formation by stabilizing synaptic connectivity. This scenario fits to the participation of SK channels reported in the studies described above. It has indeed been shown in hippocampal recordings that SK channel downregulation boosts calcium signaling and enhances the probability for LTP induction (Ngo-Anh et al., 2005).

What then are lasting roles of (SK-mediated) enhanced excitability? First, SK channels regulate dendritic plateau potentials, in particular their duration, and therefore adjust the integration of local synaptic potentials (Cai et al., 2004). Second, intrinsic plasticity may adjust the somatic spike threshold, and at least in L2/3 pyramidal neurons of the mouse S1 cortex, this effect is obtained by cholinergic co-activation (Pham and Hansel, 2023). In L2/3 pyramidal neurons of the rat barrel cortex, the resting potential sits 15–40 mV below the spike threshold, resulting in a low evoked spike rate of 0.031 spikes per whisker stimulus (Brecht et al., 2003). It is thus conceivable that threshold plasticity in individual neurons adjusts the spike threshold, resulting in circuit-specific response probabilities and spike patterns. A consequence of threshold plasticity further is that it alters how input patterns are processed. A high spike threshold is optimal for the discrimination of distinct patterns, while a reduced threshold is more easily reached when a subset of (driver) synapses are active and thus enables recognition of previously learned patterns, even when inputs are incompletely presented (Pham and Hansel, 2023). Here, too, threshold adjustment equals a shift in the optimal coding strategy and may represent an example where plasticity is meant to last at least until another event necessitates a change in strategy.

**FIGURE 5:**
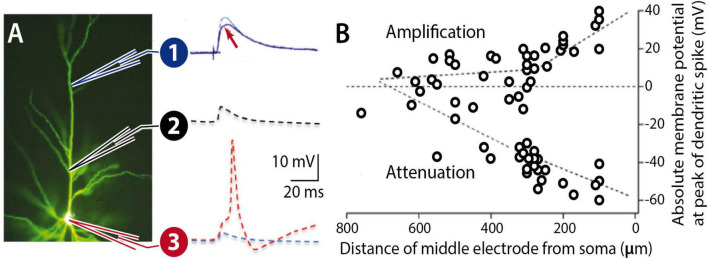
Forward propagation of dendritic EPSPs. **(A)** Pyramidal neuron filled with Lucifer Yellow. An EPSP at a distal location (1) may undergo LTP (arrow), but the dendritic potential may nevertheless experience attenuation during propagation along the proximal dendrite (2) towards the soma (3). The probability for spike firing (red) depends on the dendrite-soma coupling strength. **(B)** L5 pyramidal cell recordings show that proximal zones control forward propagation of dendritic potentials. **(B)** is adapted from Larkum et al. (2001), *J. Physiol*. 533.

While these are lasting functions / consequences of enhanced excitability and its plasticity, they do not describe an essential contribution to memory engram formation. Such an essential contribution is described in the permissive gate hypothesis. Simultaneous patch-clamp recordings from the distal dendrite, proximal dendrite and the soma of L2/3 pyramidal neurons have shown that the amplitude of distally recorded excitatory postsynaptic potentials (EPSPs) is poorly correlated with more proximal EPSP amplitudes and somatic spike output (Larkum et al., 2001; Figure 5). During propagation toward the soma, the dendritic potential is exposed to conductances that may amplify or attenuate these potentials. The density and functional availability of SK-type K^+^ conductances, for example, could regulate EPSP forward propagation and thus EPSP-spike coupling. Intrinsic plasticity may regulate the surface expression of SK channels (Ren et al., 2006) and / or their calcium sensitivity (Allen et al., 2007). It is therefore conceivable that SK channel plasticity constitutes a permissive gate for EPSP propagation.

What is the experimental evidence that supports the hypothesis that intrinsic (SK channel-mediated) plasticity gates EPSP-spike coupling and that without it LTP cannot efficiently control postsynaptic responsiveness? Recordings from cerebellar Purkinje cells have provided evidence for both claims. In somato-dendritic patch-clamp recordings from Purkinje cells *in vitro*, it was observed that either triggering intrinsic plasticity or bath-application of the SK channel antagonist apamin (10 nM) would enhance excitability and turn EPSP amplitude into a better predictor of spike output (Ohtsuki and Hansel, 2018). This finding is consistent with the first claim that SK channel plasticity modulates dendritic EPSP propagation.

**FIGURE 6:**
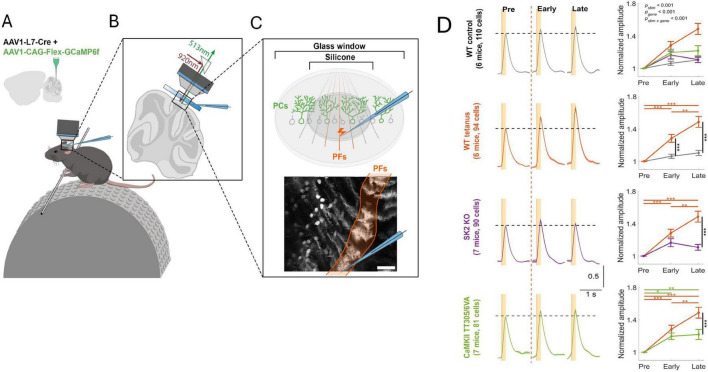
Intrinsic plasticity is an essential component of receptive field plasticity in cerebellar Purkinje cells. **(A)** Two-photon recordings from awake, head-fixed mice. AAV vectors were injected into Crus I of cerebellar cortex to express Cre-dependent GCaMP6f specifically in Purkinje cells. **(B,C)** A microelectrode was applied to stimulate a bundle of parallel fibers in Crus I. The bottom panel of **(C)** shows a representative field of view showing calcium responses in a row of Purkinje cells. In this dorsal view, each Purkinje cell is identified by a dendritic ‘stripe’ aligned in the rostrocaudal direction. The activated parallel fiber bundle is oriented perpendicularly to these dendrites. Scale bar: 100 μm. **(D)** Left: Normalized averaged calcium traces of different mouse genotypes for all trials before parallel fiber tetanization (pre; 20min), as well as during the first 20min after tetanization (early) and the subsequent 20 min (late). For tetanization, 1Hz stimulation is applied for 5min to the parallel fiber bundle. From top to bottom: WT control; WT tetanus; L7-SK2 KO tetanus; CaMKII TT305/6VA tetanus. Right: Normalized amplitude plotted over time. All data are shown as mean ± SEM. *p < 0.05; **p < 0.01, ***p < 0.001. The figure is adapted from Lin et al. (2024).

To examine whether intrinsic plasticity is essential as an enabler (permissive gate) for LTP in learning, one of us and his team tested the involvement of LTP and intrinsic plasticity, respectively, in receptive field plasticity of Purkinje cells in awake mice (Lin et al., 2024). Two-photon recordings of GCaMP6f-encoded calcium signals were used to measure the amplitude of Purkinje cell responses to parallel fiber stimulation, or tactile activation of the ipsilateral forelimb. Repeated stimulation of a parallel fiber bundle caused a potentiation of the dendritic calcium response (Figure 6). This dendritic signal and a simultaneously recorded calcium signal in the axon initial segment (AIS) are positively correlated (not shown). When the PF tetanization is applied in mice that lack SK2 channels (L7-SK2 KO), no potentiation is observed (Figure 6). When it is applied in CaMKII-TT305/6VA mice (this mutation blocks the inhibitory autophosphorylation of CaMKII; Elgersma et al., 2002), the potentiation is reduced but a significant component remains (Figure 6). In L7-SK2 KO mice, Purkinje cell intrinsic plasticity is absent, but LTP is intact (Grasselli et al., 2020). In contrast, in CaMKII-TT305/6VA mice, parallel fiber LTP is impaired, but intrinsic plasticity is intact (Belmeguenai et al., 2010; Piochon et al., 2016a). These mouse lines therefore enable an isolated impairment of LTP and intrinsic plasticity, respectively. The results of this two-photon study using awake mice suggest that both processes are needed to complete proper plasticity, but that in the absence of LTP some potentiation is still available. This is likely the result of applying a permissive gate to synapses at their given synaptic strength. Repetitive activation of tactile stimuli to a forelimb similarly potentiates dendritic responses with comparable outcomes in the two lines of genetically modified mice (Lin et al., 2024). When a PF bundle is tetanized and responses to tactile stimuli are tested before and after tetanization, response amplitudes are enhanced, and responses are even observed in cells that did not respond before tetanization. This finding shows that this type of plasticity indeed updates the receptive field of individual Purkinje cells, showing that here the interaction of synaptic and non-synaptic plasticity occurs in the context of receptive field memory in the intact animal.

## Summary and outlook

**FIGURE 7:**
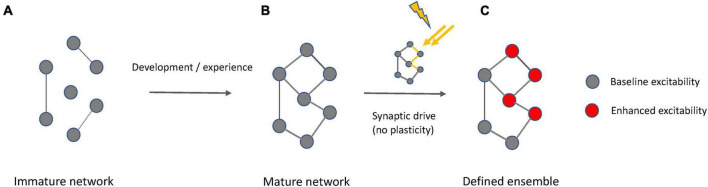
Interplay of synaptic and intrinsic mechanisms in ensemble formation. **(A)** At an immature state, a network is incompletely connected. **(B)** A mature network results from experience-dependent synapse formation and pruning during development, but also at later stages in life. Synaptic connectivity defines meaningful groups of neurons reflecting statistically relevant input relationships (e.g., in shared receptive fields). **(C)** When a mature network exists that is fully synaptically connected and encodes defined input, context-dependent activation of an ensemble based on this underlying network is possible by activity-driven enhancement of intrinsic excitability that may be transient or lasting, depending on the type of neuron and the activation conditions. This excitability enhancement requires synaptic drive but does not involve synaptic plasticity. Synaptic plasticity remains an active learning mechanism that is recruited to stabilize synapses with new information content and to adjust their input weights.

We here suggest that ensembles−including memory engrams−can be activated without significant synaptic plasticity (Figure 7). Instead, activity-dependent increases in neuronal excitability can recruit neurons into ensembles and maintain them active. There are many potential mechanisms, including cholinergic transmission, that modulate intrinsic excitability, and they are found to be quite robust and even long lasting. It is possible that this intrinsic plasticity may first occur in core neurons that anchor a wider net of participants. In this case, the core neurons could synaptically drive other participant neurons without engaging synaptic plasticity, equivalent to their own initial activation by synaptic drive, but in the strict absence of synaptic plasticity. Regardless of whether or not the intrinsic upregulation of excitability is restricted to a few core neurons or not, the underlying cellular mechanism that enables this intrinsic drive is the opening of a permissive gate that in individual neurons enhances the EPSP−spike output coupling (permissive gate theory). A permissive gate function may be executed by a variety of voltage- or calcium-activated ion channels, not just SK channels. Indeed, we stress that the SK channel work cited is but an example of what is likely to be a complex channel ecosystem that regulates neuronal excitability. Our observation that SK2 channel modulation assumes this role in both L2/3 pyramidal neurons as well as Purkinje cells underlines the role of these channels in spike burst control, but it does not exclude the participation of other channel types. In addition, permissive gate control may link functions to ensemble integration, such as reinforcement signaling (Schiess et al., 2016). The experiments described here therefore highlight only a subset of scenarios that are potentially physiologically relevant.

It is important to note that the view on ensemble activation presented here (illustrated by the iceberg model) is compatible with synaptic plasticity playing a role in circuit formation and modifications (Figure 7). The iceberg model assumes the *prior* existence of a synaptic connectivity matrix, perhaps via competitive synaptic plasticity processes and also it is consistent with the idea that synaptic weights can be adjusted in an experience-dependent manner throughout lifetime. The synaptic plasticity machinery for these processes is available from early postnatal development onwards and the molecular pathways involved in LTD and LTP remain similar across these developmental stages (Piochon et al., 2016b). When we emphasize the importance of intrinsic plasticity in ensemble and engram function, we do not suggest that synaptic plasticity is not important. Synaptic plasticity mechanisms may be the main mechanism that neurons use to detect and learn associative input relationships and establish neural circuits based on connectivity principles informed by such associative structures (Hansel, 2024). The synchronous activation of excitatory inputs, due to firing of an ensemble, could generate a “synaptoensemble”, as a group of coactive inputs that could bring postsynaptic neurons to threshold (Buzsaki, 2010). Furthermore, postsynaptic mechanisms may also contribute to gate control, particularly when changes in dendritic integration over short time periods are considered. An example are somato-dendritic gradients of chloride that change with ongoing synaptic activity and can alter the efficacy of dendritic propagation (Currin et al., 2020; see also Doyon et al., 2014; Weilinger et al., 2022). Given the specific positioning of GABAergic interneuronal input on dendritic shafts (Kwon et al., 2018), a role for this mechanism in ensemble integration needs to be further explored. However, in contrast to any synaptic mechanism, intrinsic plasticity is in principle not associative, but cell autonomous, and it is activity-dependent and reflects the activation history of a neuron. In this way, intrinsic excitability and its plasticity may act as a constant driver for drifts in neural ensemble composition and activity (Delamore et al., 2023). This view also explains why the neuronal composition of ensembles is not stable, because it keeps changing with the passing of time, since the constant barrage of neuronal activity, through intrinsic excitability, changes the circuit.

A key prediction of the model presented here is that neuronal ensembles whose activity is meaningful for brain and organismal function are signified by synaptic connectivity and enhanced excitability. Being connected is a basic requirement; it is the enhanced excitability that signifies the importance and primacy of the signal that is conveyed. Experimental and computational future work will be required to further test the model, in particular in cortical structures. This work on plasticity mechanisms needs to go hand-in-hand with attempts to better understand the nature and signaling consequences of ensembles themselves.

## Author contributions

CH: Conceptualization, Funding acquisition, Investigation, Resources, Validation, Visualization, Writing–original draft, Writing–review and editing. RY: Conceptualization, Funding acquisition, Project administration, Resources, Supervision, Validation, Visualization, Writing–original draft, Writing–review and editing.
